# Racial and ethnic disparities in a real-world precision oncology data registry

**DOI:** 10.1038/s41698-023-00351-6

**Published:** 2023-01-19

**Authors:** Alexander T. M. Cheung, Elina L. Palapattu, Isabella R. Pompa, Christopher M. Aldrighetti, Andrzej Niemierko, Henning Willers, Franklin Huang, Neha Vapiwala, Eliezer Van Allen, Sophia C. Kamran

**Affiliations:** 1 NYU Grossman School of Medicine, New York, NY USA; 2 Broad Institute of Harvard and MIT, Cambridge, MA USA; 3 Dana-Farber Cancer Institute, Boston, MA USA; 4 Department of Radiation Oncology, Massachusetts General Hospital, Harvard Medical School, Boston, MA USA; 5 Division of Hematology/Oncology, Department of Medicine, University of California, San Francisco, San Francisco, CA USA; 6 Department of Radiation Oncology, Hospital of the University of Pennsylvania, Perelman School of Medicine, Philadelphia, PA USA

**Keywords:** Prognostic markers, Health policy

## Abstract

Biorepositories enable precision oncology research by sharing clinically annotated genomic data, but it remains unknown whether these data registries reflect the true distribution of cancers in racial and ethnic minorities. Our analysis of Project Genomics Evidence Neoplasia Information Exchange (GENIE), a real-world cancer data registry designed to accelerate precision oncology discovery, indicates that minorities do not have sufficient representation, which may impact the validity of studies directly comparing mutational profiles between racial/ethnic groups and limit generalizability of biomarker discoveries to all populations.

Racial and ethnic cancer disparities persist despite tremendous strides in our understanding of cancer biology^1^. Large biorepositories created to better understand and treat cancers have historically included mostly white patients. A 2016 analysis of The Cancer Genome Atlas (TCGA) revealed all racial minority groups had insufficient representation, thus limiting our ability to confidently detect potentially actionable mutations^2^. The American Association of Cancer Research (AACR) Project Genomics Evidence Neoplasia Information Exchange (GENIE) is a large, publicly available international cancer registry populated with clinically annotated genomic sequencing information to facilitate precision medicine research^3^. Multiple groups have used Project GENIE to describe novel genomic variations between racial categories in different tumors^4–6^, yet these discoveries have conflicted with prior findings and cannot be replicated or biologically explained^7,8^. Inadequate representation of racial/ethnic minorities within Project GENIE may contribute to spurious findings and inadvertently contribute to health care disparities due to insufficient understanding of mutational findings among minority populations.

At the time of analysis, there was a total of 43,472 samples present in Project Genie by US sites only (Supplementary Table 1) and 52,773 samples among all sites (US + International sites, Supplementary Table 2). By race, white patients represented the largest proportion of GENIE samples for all cancers analyzed from the US, with a range of 67.8% (gastric) to 91.2% (melanoma) (Supplementary Table 1). White patients were adequately or over-represented with observed-to-expected ratios >1 for all cancer types except lung and melanoma (Fig. 1A, Supplementary Table 3). Asian + Pacific Islander (AAPI) patients had significant over-representation across several cancer types including colorectal (ratio 1.47, 95% CI 1.20 to 1.79, *p* < 0.001), lung (2.43, 1.80 to 3.29, *p* < 0.001), and breast (1.32, 1.10 to 1.59, *p* < 0.001) cancers, with an overall ratio of 1.46 among all cancers (95% CI 1.27 to 1.67, *p* < 0.001, Fig. 1B). Black patients were significantly under-represented in all cancers except for melanoma and cervical (statistical significance not reached due to insufficient sample size), and overall under-represented with a ratio of 0.50 (95% CI 0.40 to 0.62, *p* < 0.001, Fig. 1C). Similarly, Hispanic patients were consistently under-represented with a ratio of 0.57 (95% CI 0.50 to 0.65, *p* < 0.001) among all cancers (Fig. 1D). Notably, zero samples came from Native American patients for five cancer types, including anal, prostate, endometrial, cervical, and vaginal cancers (Supplementary Table 1); this demographic was significantly under-represented in many cancer types (Fig. 1E).

**Fig. 1:**
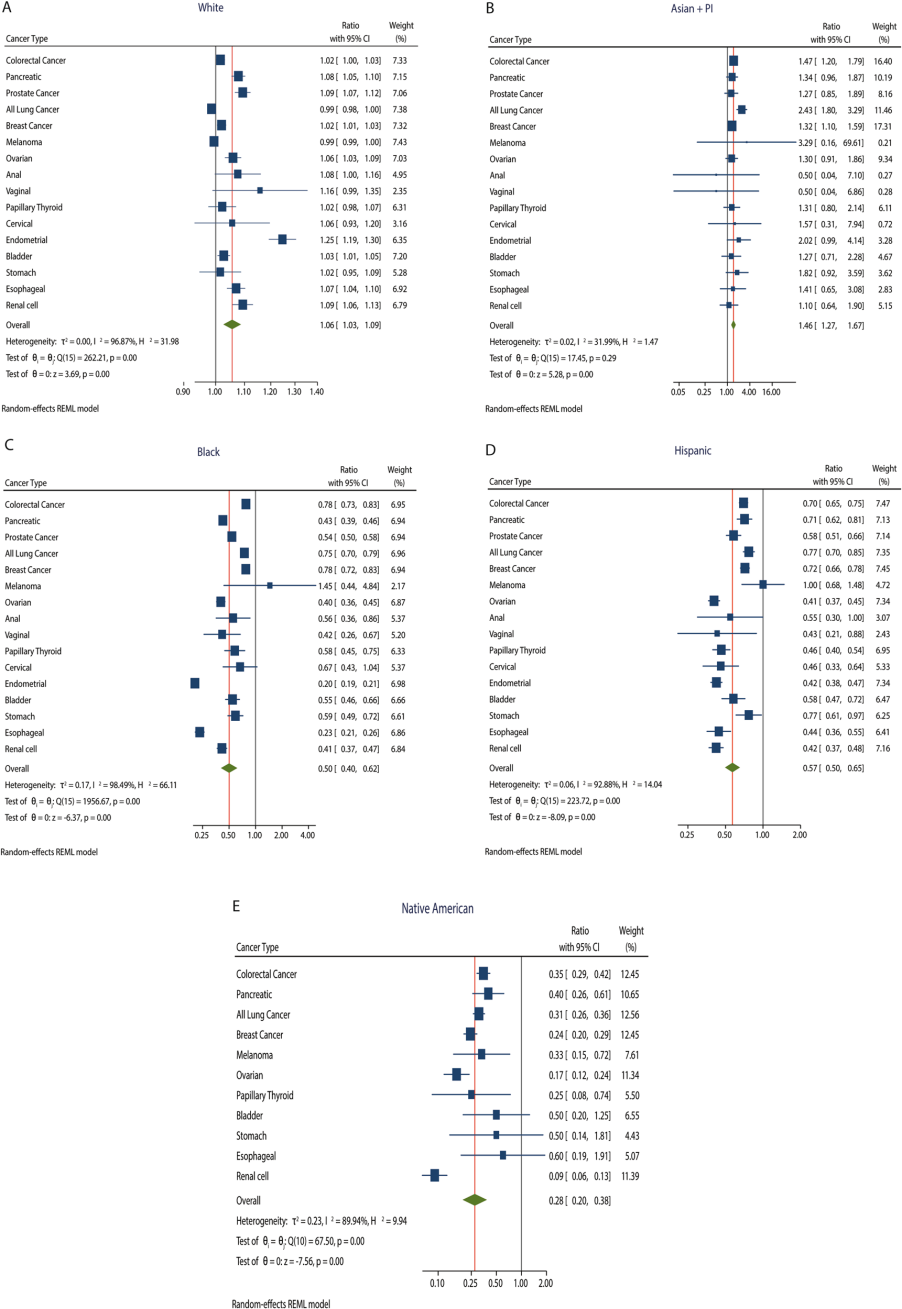
Representation of racial/ethnic samples in GENIE relative to US cancer population. Squares represent the ratio of observed to expected number of samples in GENIE; whiskers show 95% CI. Ratios to the left of the black line (1.00) indicate under-representation. Overall ratios for a given racial/ethnic group are indicated by green diamonds and red lines. **A** White. **B** Asian + PI. **C** Black. **D** Hispanic. **E** Native American.

Using the current sample landscape present in GENIE, we sought to understand whether there is sufficient power (≥0.8) to detect statistically significant mutational differences with relatively small effect sizes (Cohen’s h ≤ 0.2) between white and non-white patient samples^9^. As an example, in order to adequately power such a study for primary prostate cancer samples in GENIE, one would need 225 samples from a given non-white racial/ethnic group. Of note, no minority group in GENIE achieved such a degree of representation for this cancer type (Fig. 2A). Existing GENIE data are insufficiently powered to detect small but potentially clinically significant differences between white and non-white patients for pancreatic and prostate cancers in both the primary (Fig. 2A) and metastatic (Fig. 2B) setting. At an effect size of 0.1, no non-white racial/ethnic group had adequate representation among any of the cancer types examined to detect a difference with adequate power. Likewise, in the metastatic setting, GENIE data are currently insufficiently powered to detect small differences in proportions (0.2 effect size) at 80% power for a given mutation between white and non-white patients in all cancers except breast (Supplementary Table 4, Fig. 2B), yet this disappeared at an effect size of 0.15. GENIE was not sufficiently powered to detect differences between white and Native American or Pacific Islander patients in any of the five cancers analyzed, for either primary or metastatic tumors.

**Fig. 2:**
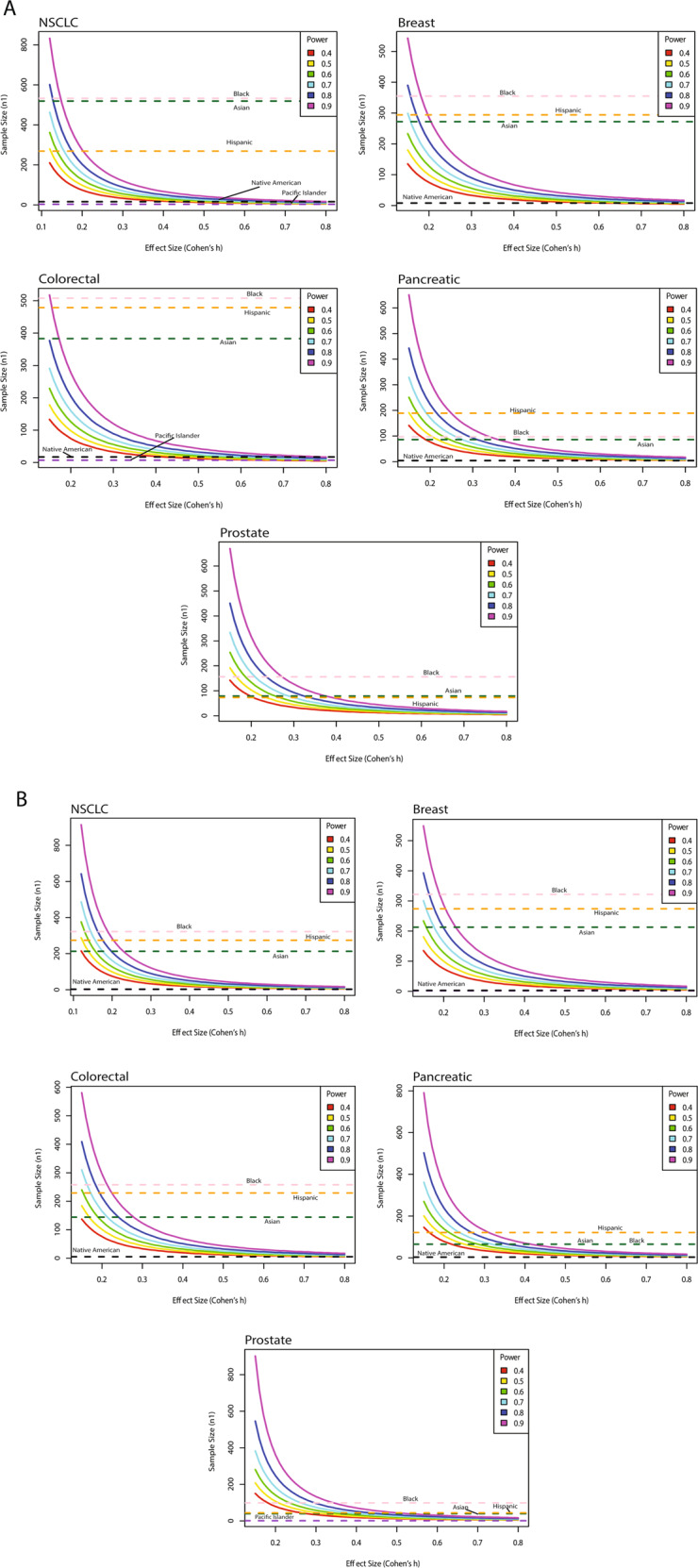
Sample size estimation to detect differences in mutational proportions between white and racial/ethnic minority patients at varying power increments. Estimated number of samples (y-axis, n1) required to detect differences in mutational proportions between white and non-white patients at varying statistical power (solid curved lines) for different effect sizes (x-axis). All horizontal dashed lines indicate the current actual number of samples in GENIE for a given racial/ethnic group. Black (dashed pink), Hispanic (dashed orange), Asian (dashed dark green), Native American (dashed black), and Pacific Islander (dashed purple) racial/ethnic groups are displayed. Power = 0.8 is represented by the solid dark blue line. **A** Primary Cancer. **B** Metastatic Cancer.

Project GENIE provides public access to a rich genomics biorepository that includes many cancer types to democratize and accelerate precision oncology research. However, our findings suggest that these data do not reflect the true landscape of cancer patients in the US and thus may misrepresent the disease burden in many racial/ethnic minority populations. White patients are adequately or over-represented across nearly all cancer types within GENIE, while the opposite is true for most minorities. The only non-white racial/ethnic group to have over-representation in any cancer type was the AAPI category, yet even these results are confounded. “Asian” refers to a highly heterogeneous group in both ancestral and socioeconomic metrics^10^, and the inclusion of geographically and culturally distinct groups like Native Hawaiians only further confounds meaningful analysis. Moreover, even though ancestry provides a more precise estimate of genetic risk than does race, researchers should never conflate either of these as a proxy for social or environmental circumstance^11^. Race is a social construct that can vary by generation and geographic location, while ancestry alone provides no information regarding the external influences on an individual’s health.

Our findings further suggest that GENIE is not sufficiently powered to detect small yet potentially clinically meaningful differences between white and non-white patients in even the most common cancer types. We hope that these results spur recruitment efforts that are targeted towards the deficits in representation we have identified and commensurate with the number of minority samples needed to adequately power future studies (see Fig. 2A, B). In particular, groups such as Native Americans and Pacific Islanders (after disaggregation from AAPI) have zero representation in many cancer types, which threatens to exacerbate cancer disparities.

While unfortunate, these results reflect the historical status quo for precision medicine research. A 2009 evaluation of genome-wide association studies demonstrated that 96% of participants were of European descent^12^. As recently as 2021, a review of precision oncology clinical trials revealed consistent under-representation of non-white participants^13^. The reasons for low representation are likely multifactorial, ranging from institutional exclusion to minority mistrust of research agendas, yet the imperative remains: diversity ought be a mission-critical priority for precision medicine^12^. Thus, any precision medicine breakthroughs or findings from this biorepository may not be broadly applicable to, or safe for, our diverse cancer populations.

It is important to note that genomics represents only one aspect of cancer as an illness, despite its status as a “disease of the genome.”^14^ Social determinants, environment, and prior treatments can influence the somatic mutational profile of patients and their response to subsequent therapies^15^. Our analysis could not account for factors that may have biased enrollment of GENIE participants, such as zip code or educational levels, due to a lack of documentation in the database. In addition, despite possible mutational differences between races/ethnicities, research demonstrates that factors such as healthcare access and other social determinants of health may contribute a larger role to observed differences related to treatment and clinical outcomes.

While we have discussed the issue of representation in datasets like GENIE from cancer genomics viewpoint, we recognize that there are many other disciplines that should contribute to this complex and multi-faceted issue. In fact, some have argued geneticists have monopolized the discussion of diversity, while sociologists, ethicists, and political scientists deserve a stronger voice given their historical expertise^16,17^. Processes such as deliberative democracy that engage research populations to elicit their perspectives, hopes, or misconceptions regarding research agendas can help to foster trust^18,19^. However, we believe even basic changes in methodology and recruitment for future biorepositories can dramatically improve their ability to address health disparities. For one, standardized methods to obtain demographic information of participants should be implemented among all participating institutions and self-identified ethnicities ought to have more options than the binary “Hispanic” or “Non-Hispanic” present in the current GENIE database. We also advocate for the incorporation of social determinants of health, careful annotation of prior treatments, and other patient-related information in future datasets so unique discoveries or associations can be better contextualized and understood. Lastly, stakeholders must advocate for such changes and hold their findings to higher standards when utilizing incomplete or imperfect datasets so that precision oncology is equitable and accessible.

This study was limited by the aggregation of several cancer types into one label (e.g., “Lung”) to compare across datasets (US cancer incidence and GENIE), and several patients had no race/ethnicity information—although this was a small proportion of the total dataset (8.6%). In addition, it is not known how race/ethnicity is assessed or captured, such as by self-report or deteremined by a researcher, and whether this is standardized across institutions. We also assumed that the Centers for Disease Control and Prevention (CDC) dataset used to define the expected proportion of cancer patients (see Methods: Statistical Analysis) is truly representative of the US cancer population.

Our analysis demonstrates that the Project GENIE database, while a valuable tool for oncology researchers, under-represents racial/ethnic minorities in several cancer types and should be used cautiously in studies that directly compare racial/ethnic groups. It is imperative that we advance precision oncology discoveries beyond academic settings and out into the community, starting with using datasets that contain patients who reflect our broad cancer community populations.

## Methods

Clinical and genomic data were downloaded from the AACR Project GENIE repository (v9.1) on synapse.org in September 2021 for 17 cancers identified by OncoTree Code (Supplementary Table 5). Analysis was performed September 2021-July 2022. Cancer types were evaluated if there were at minimum 75 samples present in the GENIE repository at time of analysis and focused on solid tumor types. Data were categorized by the six racial/ethnic groups as reported in GENIE (white, Black, Asian, Pacific Islander, Native American, and Hispanic) and by primary versus metastatic tumor types. Patients with multiple samples were only counted once when calculating the overall demographic characteristics of GENIE by cancer type (Supplementary Table 1). In addition, patients with both primary and metastatic samples were counted once for each respective category as these analyses were independent of one another. This study was determined to be exempt from human participant research guidelines because it was a secondary analysis of publicly available published reports and data by the Mass General Brigham institutional review board.

### Statistical analysis

To understand the current GENIE tumor sample landscape relative to the broader cancer population, we utilized the Centers for Disease Control and Prevention Wide-ranging Online Data for Epidemiologic Research (CDC WONDER) database to define the proportion of cancer patients we would expect to see from each racial/ethnic group in a truly representative dataset^20^. For example, if Black patients made up 20% of a given cancer type in CDC WONDER (the “expected proportion”), approximately 20% of GENIE samples for that same cancer type should likewise represent Black participants. The “expected” number of samples from each racial/ethnic group was then estimated by multiplying the number of US-based GENIE samples for a given cancer type (Supplementary Table 6) by the expected proportion of each group for that given cancer. We defined “representation” as the ratio of the actual number of GENIE samples to the expected number of samples per cancer type, with 95% exact binomial confidence intervals (CI) estimated. A statistically significant ratio >1 indicated over-representation, while a ratio <1 indicated under-representation. A random-effects meta-analysis of under-representation and over-representation ratios of individual cancers (relative to the US-based proportion of racial and ethnic groups for a given cancer type) was performed.

At the time of analysis (September 2021), 2017 cancer incidence rates were available. Age-standardized incidence rates adjusted to the 2000 standard US population by race and ethnicity were used to calculate cancer cases as a measure of proportion of cancer burden by race and ethnicity. We could not normalize GENIE data by median year of enrollment to determine incidence as we did not have the year a sample was added to the AACR GENIE database. US cancer incidence databases do not distinguish “Asian” from “Pacific Islander”, thus GENIE samples from these groups were combined (“AAPI”) for direct comparison. In addition, GENIE only included “Spanish/Hispanic” or “Non-Spanish/non-Hispanic” as ethnic categories, where the latter group could include a range of racial categories. Results are presented using forest plots showing cancer type-specific effect sizes, and overall effect size for each racial and ethnic group, with respective 95% confidence intervals. Test statistics for the pooled data and estimates for between-cancer type heterogeneity statistics are also shown. Data were analyzed using Stata version 17 (StataCorp).

To estimate the number of non-white racial/ethnic samples needed to detect differences in mutational proportions with sufficient power when directly compared to current number of white patient samples (Supplementary Table 4) using all participating centers in GENIE (Supplementary Table 2), a simulation experiment^21^ was performed using the Rv4.1.2 package “pwr.”^22^ Analysis was limited to prioritize the five deadliest U.S. cancer types (non-small cell lung cancer [NSCLC], breast, colorectal, pancreatic, and prostate) in the primary (Supplementary Table 7) and metastatic (Supplementary Table 8) setting using data from all participating centers (US + International). The number of samples was determined at varying Cohen’s h (*Cohen’s h* = *2 arcsin √*p_*1*_ *–* *2 arcsin √*p_*2*_*)* proportional difference effect sizes for various power increments, at a *p* = 0.05 significance level^9^. The effect size was approximated as “small” if *h* = 0.2, “medium” if *h* = 0.5, and “large” if *h* = 0.8. This analysis did not require US cancer incidence data and was therefore able to distinguish Asian from Pacific Islander. A small proportional effect size difference (e.g. *h* ≤ 0.2) of a given mutation between white and non-white cohorts may be clinically significant, particularly if the mutation of interest is clinically actionable.

### Reporting summary

Further information on research design is available in the Nature Research Reporting Summary linked to this article.

### Supplementary information

REPORTING SUMMARY

Supplemental Tables

## Data Availability

The data underlying this article are available via the publicly available Project GENIE at genie.cbioportal.com, with additional information regarding its initial release found here: 10.1158/2159-8290.CD-17-0151.
