# Supplementary material for: Racial and ethnic disparities in a real-world precision oncology data registry
Source: NPJ Precis Oncol. 2023 Jan 19;7:7. doi: 10.1038/s41698-023-00351-6 (PMC9852424; doi:10.1038/s41698-023-00351-6)
Supplement: Supplementary file 1 — REPORTING SUMMARY [file 41698_2023_351_MOESM1_ESM.pdf]

nature portfolio

Corresponding author(s): Sophia C. Kamran MD

Last updated by author(s): Nov 16, 2022

Reporting Summary

Nature Portfolio wishes to improve the reproducibility of the work that we publish. This form provides structure for consistency and transparency in reporting. For further information on Nature Portfolio policies, see our Editorial Policies and the Editorial Policy Checklist.

Statistics

For all statistical analyses, confirm that the following items are present in the figure legend, table legend, main text, or Methods section.

n/a	Confirmed	
☐	☒	The exact sample size (*n*) for each experimental group/condition, given as a discrete number and unit of measurement
☐	☒	A statement on whether measurements were taken from distinct samples or whether the same sample was measured repeatedly
☐	☒	The statistical test(s) used AND whether they are one- or two-sided<br>*Only common tests should be described solely by name; describe more complex techniques in the Methods section.*
☒	☐	A description of all covariates tested
☒	☐	A description of any assumptions or corrections, such as tests of normality and adjustment for multiple comparisons
☐	☒	A full description of the statistical parameters including central tendency (e.g. means) or other basic estimates (e.g. regression coefficient) AND variation (e.g. standard deviation) or associated estimates of uncertainty (e.g. confidence intervals)
☐	☒	For null hypothesis testing, the test statistic (e.g. *F*, *t*, *r*) with confidence intervals, effect sizes, degrees of freedom and *P* value noted<br>*Give P values as exact values whenever suitable.*
☒	☐	For Bayesian analysis, information on the choice of priors and Markov chain Monte Carlo settings
☒	☐	For hierarchical and complex designs, identification of the appropriate level for tests and full reporting of outcomes
☐	☒	Estimates of effect sizes (e.g. Cohen's *d*, Pearson's *r*), indicating how they were calculated

Our web collection on statistics for biologists contains articles on many of the points above.

Software and code

Policy information about availability of computer code

Data collection	The data underlying this article are available via the publicly available Project GENIE at genie.cbioportal.com, with additional information regarding its initial release found here: https://doi.org/10.1158/2159-8290.CD-17-0151.
Data analysis	Data were analyzed using Stata version 17 (StataCorp) as well as Rv4.1.2

For manuscripts utilizing custom algorithms or software that are central to the research but not yet described in published literature, software must be made available to editors and reviewers. We strongly encourage code deposition in a community repository (e.g. GitHub). See the Nature Portfolio guidelines for submitting code & software for further information.

Data

Policy information about availability of data

All manuscripts must include a data availability statement. This statement should provide the following information, where applicable:

- Accession codes, unique identifiers, or web links for publicly available datasets
- A description of any restrictions on data availability
- For clinical datasets or third party data, please ensure that the statement adheres to our policy

The data underlying this article are available via the publicly available Project GENIE at genie.cbioportal.com, with additional information regarding its initial release found here: https://doi.org/10.1158/2159-8290.CD-17-0151.

Human research participants

Policy information about studies involving human research participants and Sex and Gender in Research.

Reporting on sex and gender	This information was not collected or analyzed. Our focus was on race/ethnicity-based analyses and thus the data was not further disaggregated by sex/gender categories.
Population characteristics	Clinical and genomic data were downloaded from the AACR Project GENIE repository (v9.1) on synapse.org in September 2021 for 17 cancers identified by OncoTree Code (ST5). Analysis was performed September 2021-July 2022. Cancer types were evaluated if there were at minimum 75 samples present in the GENIE repository at time of analysis and focused on solid tumor types. Data were categorized by the six racial/ethnic groups as reported in GENIE (White, Black, Asian, Pacific Islander, Native American, and Hispanic) and by primary versus metastatic tumor types.
Recruitment	Patients were not recruited to this study- we used publicly available published reports and data present in genie.cBioPortal.com. Patient data and information was present in this public database.
Ethics oversight	MGB

Note that full information on the approval of the study protocol must also be provided in the manuscript.

Field-specific reporting

Please select the one below that is the best fit for your research. If you are not sure, read the appropriate sections before making your selection.

☒ Life sciences ☐ Behavioural & social sciences ☐ Ecological, evolutionary & environmental sciences

For a reference copy of the document with all sections, see nature.com/documents/nr-reporting-summary-flat.pdf

Life sciences study design

All studies must disclose on these points even when the disclosure is negative.

Sample size	Sample sizes were not selected- we used the number of samples available in the GENIE database for each cancer type at the time of the analysis. We then performed a power calculation study to determine whether the number of samples in a non-White race/ethnicity were adequate to compare to White individuals for an effect size based on a simulation experiment for a mutation.
Data exclusions	Cancer types were evaluated if there were at minimum 75 samples present in the GENIE repository at time of analysis and focused on solid tumor types.
Replication	NA
Randomization	Samples were grouped by cancer type and race/ethnicity.
Blinding	Blinding was not relevant to this study.

Reporting for specific materials, systems and methods

We require information from authors about some types of materials, experimental systems and methods used in many studies. Here, indicate whether each material, system or method listed is relevant to your study. If you are not sure if a list item applies to your research, read the appropriate section before selecting a response.

Materials & experimental systems

n/a	Involved in the study	
☒	☐	Antibodies
☒	☐	Eukaryotic cell lines
☒	☐	Palaeontology and archaeology
☒	☐	Animals and other organisms
☐	☒	Clinical data
☒	☐	Dual use research of concern

Methods

n/a	Involved in the study	
☒	☐	ChIP-seq
☒	☐	Flow cytometry
☒	☐	MRI-based neuroimaging

Clinical data

Policy information about clinical studies

All manuscripts should comply with the ICMJE guidelines for publication of clinical research and a completed CONSORT checklist must be included with all submissions.

Clinical trial registration	No clinical trial registration for this study.
Study protocol	NA- This study was determined to be exempt from human participant research guidelines because it was a secondary analysis of publicly available published reports and data by the Mass General Brigham institutional review board.
Data collection	Clinical and genomic data were downloaded from the AACR Project GENIE repository (v9.1) on synapse.org in September 2021 for 17 cancers identified by OncoTree Code. Analysis was performed September 2021-July 2022
Outcomes	To understand the current GENIE tumor sample landscape relative to the broader cancer population, we utilized the Centers for Disease Control and Prevention Wide-ranging Online Data for Epidemiologic Research (CDC WONDER) database to define the proportion of cancer patients we would expect to see from each racial/ethnic group in a truly representative dataset. We defined "representation" as the ratio of the actual number of GENIE samples to the expected number of samples per cancer type, with 95% exact binomial confidence intervals (CI) estimated. A statistically significant ratio >1 indicated over-representation, while a ratio <1 indicated under-representation. A random-effects meta-analysis of under-representation and over-representation ratios of individual cancers (relative to the US-based proportion of racial and ethnic groups for a given cancer type) was performed. To estimate the number of non-White racial/ethnic samples needed to detect differences in mutational proportions with sufficient power when directly compared to current number of White patient samples using all participating centers in GENIE, a simulation experiment was performed using the Rv4.1.2 package "pwr." Analysis was limited to prioritize the five deadliest U.S. cancer types (non-small cell lung cancer [NSCLC], breast, colorectal, pancreatic, and prostate) in the primary and metastatic setting using data from all participating centers (US + International). The number of samples was determined at varying Cohen's h (Cohen's h=2 arcsin √p1 – 2 arcsin √p2) proportional difference effect sizes for various power increments, at a p=0.05 significance level. The effect size was approximated as "small" if h = 0.2, "medium" if h = 0.5, and "large" if h = 0.8.
