# Supplementary material for: Racial and ethnic disparities in a real-world precision oncology data registry
Source: NPJ Precis Oncol. 2023 Jan 19;7:7. doi: 10.1038/s41698-023-00351-6 (PMC9852424; doi:10.1038/s41698-023-00351-6)
Supplement: Supplementary file 2 — Supplemental Tables [file 41698_2023_351_MOESM2_ESM.pdf]

Racial and Ethnic Disparities in a Real-World Precision Oncology Data Registry

Alexander TM Cheung MBHL*[1,2], Elina L. Palapattu*[2,3], Isabella R. Pompa BA[4], Christopher M. Aldrighetti BS[4], Andrzej Niemierko PhD[4], Henning Willers MD[4], Franklin Huang MD PhD[5], Neha Vapiwala MD[6], Eli Van Allen MD[2,3], Sophia C. Kamran MD[ǂ 2,4]

*co-first authors; ǂcorresponding author

1. NYU Grossman School of Medicine, New York, NY
2. Broad Institute of Harvard and MIT, Cambridge, MA
3. Dana-Farber Cancer Institute, Boston, MA
4. Department of Radiation Oncology, Massachusetts General Hospital, Harvard Medical School, Boston, MA
5. Division of Hematology/Oncology, Department of Medicine, University of California, San Francisco, San Francisco, CA
6. Department of Radiation Oncology, Hospital of the University of Pennsylvania, Perelman School of Medicine, Philadelphia, PA

Table of contents

1. **Supplemental Tables…………………………………………………………………..Pg. 2-9**

Supplemental Table 1 (ST1). Demographic Characteristics of Cancers in AACR GENIE: USA Sites Only

Characteristic	Thyroid	Melanoma	*NSCLC	^SCLC	Breast	Esophageal	Stomach	Pancreatic	Colorectal	Anal	Bladder	#RCC	Prostate	Ovarian	Endometrial	Cervical	Vaginal
Total Number	705	2799	11384	505	7907	1002	587	3056	8607	173	1686	1249	2879	1897	1674	166	75
Race (n%)																	
White	561 (79.6%)	2552 (91.2%)	8798 (77.3%)	422 (83.6%)	6032 (76.3%)	899 (88.7%)	398 (67.8%)	2544 (83.2%)	6519 (75.7%)	156 (90.2%)	1475 (87.5%)	1027 (82.2%)	2349 (81.6%)	1577 (83.1%)	1358 (81.1%)	113 (68.1%)	66 (88.0%)
Black	29 (4.1%)	16 (0.6%)	858 (7.5%)	14 (2.8%)	655 (8.3%)	19 (1.9%)	49 (8.3%)	153 (5.0%)	743 (8.6%)	10 (5.8%)	52 (3.1%)	56 (4.5%)	239 (8.3%)	68 (3.6%)	77 (4.6%)	14 (8.4%)	5 (6.7%)
Asian+ Pacific Islander	72 (8.4%)	23 (0.8%)	771 (6.8%)	24 (4.8%)	433 (5.5%)	31 (3.1%)	7 (12.1%)	135 (4.4%)	477 (5.5%)	1 (0.6%)	42 (2.5%)	33 (2.6%)	85 (3.0%)	108 (5.7%)	89 (5.3%)	11 (6.6%)	1 (1.3%)
Native American	1 (0.1%)	2 (0.1%)	20 (0.2%)	1 (0.2%)	10 (0.1%)	3 (0.3%)	2 (0.3%)	6 (0.2%)	22 (0.2%)	0 (0%)	3 (0.2%)	1 (0.1%)	0 (0%)	2 (0.1%)	0 (0%)	0 (0%)	0 (0%)
NA	55 (7.8%)	206 (7.4%)	937 (8.2%)	44 (8.7%)	777 (9.8%)	60 (6.0%)	67 (11.4%)	218 (7.1%)	847 (9.7%)	6 (3.5%)	114 (6.8%)	132 (10.6%)	206 (7.2%)	142 (7.5%)	150 (9.0%)	28 (16.9%)	3 (4.0%)
Ethnicity (n%)																	
Hispanic	44 (6.2%)	50 (1.8%)	351 (3.1%)	16 (3.2%)	448 (5.7%)	23 (2.3%)	63 (10.7%)	167 (5.5%)	477 (5.5%)	6 (3.5%)	44 (2.6%)	53 (4.2%)	108 (3.8%)	79 (4.2%)	85 (5.1%)	11 (6.6%)	3 (4.0%)
Non-Hispanic	622 (88.2%)	2203 (78.7%)	9796 (86.1%)	452 (89.5%)	6717 (85.0%)	895 (89.3%)	473 (80.6%)	2673 (87.5%)	6786 (78.8%)	148 (85.5%)	1557 (92.3%)	1102 (88.2%)	2577 (89.5%)	1699 (89.6%)	1505 (89.9%)	131 (78.9%)	70 (93.3%)
NA	39 (5.5%)	546 (19.5%)	1237 (10.9%)	37 (7.3%)	742 (9.4%)	84 (8.4%)	51 (8.7%)	216 (7.1%)	1344 (15.6%)	19 (11.0%)	85 (5.0%)	94 (7.5%)	194 (6.7%)	119 (6.3%)	84 (5.0%)	24 (14.5%)	2 (2.7%)

*NSCLC= non-small cell lung cancer
^SCLC = small cell lung cancer
[#]RCC= renal cell carcinoma

Supplemental Table 2 (ST2). Demographic Characteristics of Cancers in AACR GENIE: All Sites

Characteristic	Thyroid	Melanoma	*NSCLC	^SCLC	Breast	Esophageal	Stomach	Pancreatic	Colorectal	Anal	Bladder	#RCC	Prostate	Ovarian	Endometrial	Cervical	Vaginal
Total Number	771	3074	12351	519	10651	1071	625	3335	9576	191	1787	1317	3022	2338	1839	213	93
Race (n%)																	
White	595 (77.2%)	2672 (87%)	9223 (74.7%)	424 (81.7%)	6348 (59.6%)	919 (85.8%)	417 (66.7%)	2758 (82.7%)	7050 (73.6%)	163 (85.3%)	1522 (85.2%)	1063 (80.7%)	2403 (79.5%)	1897 (81.1%)	1468 (79.8%)	138 (64.8%)	78 (83.9%)
Black	32 (4.2%)	16 (0.5%)	873 (7.1%)	14 (2.7%)	661 (6.2%)	19 (1.8%)	49 (7.8%)	159 (4.8%)	758 (7.9%)	10 (5.2%)	53 (3.0%)	58 (4.4%)	243 (8%)	77 (3.3%)	82 (4.5%)	14 (6.6%)	6 (6.5%)
Asian	72 (9.3%)	24 (0.8%)	811 (6.6%)	26 (5%)	470 (4.4%)	33 (3.1%)	72 (11.5%)	148 (4.4%)	514 (5.4%)	2 (1%)	50 (2.8%)	34 (2.6%)	87 (2.88%)	159 (6.8%)	108 (5.9%)	14 (6.6%)	4 (4.3%)
Pacific Islander	0 (0%)	0 (0%)	7 (0.06%)	0 (0%)	2 (0.02%)	0 (0%)	0 (0%)	0 (0%)	7 (0.07%)	0 (0%)	0 (0%)	0 (0%)	1 (0.03%)	3 (0.1%)	1 (0.05%)	0 (0%)	0 (0%)
Native American	1 (0.1%)	2 (0.1%)	22 (0.2%)	1 (0.2%)	11 (0.1%)	3 (0.3%)	2 (0.3%)	7 (0.2%)	22 (0.23%)	0 (0%)	3 (0.2%)	1 (0%)	0 (0%)	2 (0.1%)	0 (0%)	1 (0.5%)	0 (0%)
NA	71 (9.2%)	360 (11.7%)	1415 (11.5%)	54 (10.4%)	3159 (29.7%)	97 (9.1%)	85 (13.6%)	263 (7.9%)	1225 (12.8%)	16 (8.4%)	159 (8.9%)	161 (12.2%)	288 (9.5%)	200 (8.6%)	180 (9.8%)	46 (21.6%)	5 (5.4%)
Ethnicity (n%)																	
Hispanic	47 (6.1%)	90 (2.9%)	419 (3.4%)	18 (3.5%)	554 (5.2%)	35 (3.3%)	72 (11.5%)	307 (9.2%)	697 (7.3%)	9 (4.7%)	48 (2.7%)	70 (5.3%)	113 (3.7%)	119 (5.1%)	107 (5.8%)	13 (6.1%)	6 (6.5%)
Non-Hispanic	622 (80.7%)	2235 (72.7%)	10097 (81.7%)	453 (87.3%)	6734 (63.2%)	895 (83.7%)	473 (75.7%)	2699 (81%)	6870 (71.7%)	148 (77.5%)	1559 (87.2%)	1105 (83.9%)	2577 (85.3%)	1700 (72.7%)	1506 (81.9%)	131 (61.5%)	71 (76.3%)
NA	102 (13.2%)	749 (24.4%)	1835 (14.9%)	48 (9.2%)	3363 (31.6%)	141 (13.2%)	80 (12.8%)	329 (9.8%)	2009 (21%)	34 (17.8%)	180 (10.1%)	142 (10.8%)	332 (11%)	519 (22.2%)	226 (12.3%)	69 (32.4%)	16 (17.2%)

*NSCLC= non-small cell lung cancer
^SCLC = small cell lung cancer
#RCC= renal cell carcinoma

Supplemental Table 3 (ST3). Observed/Expected Ratio of Samples by Cancer Type in AACR GENIE US-only institutions as compared to US Cancer Population

	Observed/Expected ratio (95% CI)				
	**White**	**Asian + Pacific Islander (AAPI)**	**Black**	**Hispanic**	**Native American**
**Colorectal**	1.02* (1.00-1.03)	1.47* (1.20-1.79)	0.78* (0.73-0.83)	0.70* (0.65-0.75)	0.35* (0.91-0.42)
**Pancreatic**	1.08* (1.05-1.10)	1.34 (0.96-1.87)	0.43* (0.39-0.46)	0.71* (0.62-0.81)	0.40* (0.26-0.61)
**Prostate**	1.09* (1.07-1.12)	1.27 (0.85-1.89)	0.54* (0.50-0.58)	0.58* (0.51-0.66)	0
**Lung**	0.99 (0.98-1.00)	2.43* (1.80-3.29)	0.75* (0.70-0.79)	0.77* (0.70-0.85)	0.31* (0.26-0.36)
**Breast**	1.02* (1.01-1.03)	1.32* (1.10-1.59)	0.78* (0.72-0.83)	0.72* (0.66-0.78)	0.24* (0.20-0.29)
**Melanoma**	0.99 (0.99-1.00)	3.29 (0.16-69.61)	1.45 (0.44-4.84)	1.00 (0.68-1.48)	0.33* (0.15-0.72)
**Ovarian**	1.06* (1.03-1.09)	1.30 (0.91-1.86)	0.40* (0.36-0.45)	0.41* (0.37-0.45)	0.17* (0.12-0.24)
**Anal**	1.08 (1.00-1.16)	0.50 (0.04-7.10)	0.56* (0.36-0.86)	0.55 (0.30-1.00)	0
**Vaginal**	1.16 (0.99-1.35)	0.50 (0.04-6.86)	0.42* (0.26-0.67)	0.43* (0.21-0.88)	0
**Thyroid**	1.02 (0.98-1.07)	1.31 (0.80-2.14)	0.58* (0.45-0.75)	0.46* (0.40-0.54)	0.25* (0.08-0.74)
**Cervical**	1.06 (0.93-1.20)	1.57 (0.31-7.94)	0.67 (0.43-1.04)	0.46* (0.33-0.64)	0
**Endometrial**	1.25* (1.19-1.30)	2.02 (0.99-4.14)	0.20* (0.19-0.21)	0.42* (0.38-0.47)	0
**Bladder**	1.03* (1.01-1.05)	1.27 (0.71-2.28)	0.55* (0.46-0.66)	0.58* (0.47-0.72)	0.50 (0.20-1.25)
**Stomach**	1.02 (0.95-1.09)	1.82 (0.92-3.59)	0.59* (0.49-0.72)	0.77* (0.61-0.97)	0.50 (0.14-1.81)
**Esophagus**	1.07* (1.04-1.10)	1.41 (0.65-3.08)	0.23* (0.21-0.26)	0.44* (0.36-0.55)	0.60 (0.19-1.91)
**Renal Cell Carcinoma**	1.09* (1.06-1.13)	1.10 (0.64-1.90)	0.41* (0.37-0.62)	0.42* (0.37-0.48)	0.09* (0.06-0.13)
**Overall**	1.06* (1.03-1.09)	1.46* (1.27-1.67)	0.50* (0.40-0.62)	0.57*(0.50-0.65)	0.28* (0.20-0.38)

*$p<0.05$

Supplemental Table 4 (ST4). Number of samples needed to detect differences in mutational proportions at 80% power in comparison to white patient samples at small effect size

	Current number of white samples in database (n)	Number (n) of samples needed for 80% power at a given effect size for p=0.05 significance level			Current number of non-white samples in database by race/ethnic group (n)				
		Cohen's h effect size							
		h=0.1	h=0.15	h=0.2	Black	Asian	Hispanic	Pacific Islander	Native American
Primary									
**NSCLC***	5898	906	371	203	533	519	269	3	16
**Breast**	3359	1025	390	209	355	272	294	0	8
**Colorectal**	4788	939	377	205	508	383	479	7	17
**Pancreatic**	1653	1495	443	223	97	86	189	0	4
**Prostate**	1545	1596	451	225	156	49	73	0	0
Metastatic									
**NSCLC***	3622	1003	387	208	367	351	168	4	7
**Breast**	3157	1045	393	210	322	213	274	2	3
**Colorectal**	2389	1169	409	214	258	144	229	0	5
**Pancreatic**	1141	2515	503	237	65	65	121	0	3
**Prostate**	969	4131	546	247	99	40	44	1	0

*NSCLC= non-small cell lung cancer

Supplemental Table 5 (ST5). OncoTree Codes for Each Cancer

Cancer Type	OncoTree Code
Thyroid	THPA
Melanoma	SKCM, MEL, MUP
*NSCLC	NSCLC, LUAD, LUSC
^SCLC	SCLC
Breast	IDC, ILC, BRCA, MDLC, BRCNOS, BRCANOS
Esophageal	ESCA, ESCC
Stomach	STAD
Pancreatic	PAAD
Colorectal	COAD, COADREAD, READ
Anal	ANSC
Bladder	BLCA
#RCC	CCRCC, RCC, PRCC, URCC, CHRCC
Prostate	PRAD
Ovarian	HGSOC, SOC
Endometrial	UCEC, UEC
Cervical	CESC
Vaginal	VSC

*NSCLC= non-small cell lung cancer
^SCLC = small cell lung cancer
[#]RCC= renal cell carcinoma

Supplemental Table 6 (ST6). AACR GENIE Participating Centers

**Abbreviation**	**Center**
NKI*	Netherlands Cancer Institute, on behalf of the Center for Personalized Cancer Treatment, Amsterdam, Netherlands
DFCI	Dana-Farber Cancer Institute, Boston, MA, USA
GRCC*	Institut Gustave Roussy, Paris, France
JHU	Johns Hopkins Sidney Kimmel Comprehensive Cancer Center, Baltimore, MD, USA
MSK	Memorial Sloan Kettering Cancer Center, New York, NY, USA
UHN*	Princess Margaret Cancer Centre, University Health Network, Toronto, Ontario, Canada
MDA	The University of Texas MD Anderson Cancer Center, Houston, TX, USA
VICC	Vanderbilt-Ingram Cancer Center, Nashville, TN, USA
CRUK*	Cancer Research UK Cambridge Centre, University of Cambridge, Cambridge, England
CHOP	Children’s Hospital of Philadelphia, Philadelphia, PA, USA
DUKE	Duke Cancer Institute, Duke University Health System, Durham, NC, USA
COLU	The Herbert Irving Comprehensive Cancer Center, Columbia University, New York, NY, USA
SCI	Swedish Cancer Institute, Seattle, WA, USA
UCSF	University of California, San Francisco, CA, USA
VHIO*	Vall d’Hebron Institute of Oncology, Barcelona, Spain
WAKE	Wake Forest Baptist Medical Center, Wake Forest University Health Sciences, Winston-Salem, NC, USA
YALE	Yale Cancer Center, Yale University, New Haven, Connecticut, USA
UCHI	University of Chicago Comprehensive Cancer Center, Chicago, IL, USA

*Non-USA institutions

Supplemental Table 7 (ST7). Demographic Characteristics of Select Primary Cancers in AACR GENIE: All Sites

Characteristic	*NSCLC	Breast	Colorectal	Pancreatic	Prostate
Total Number	7803	6658	6448	1981	1887
Race					
White	5898	3359	4788	1653	1545
Black	533	355	508	97	156
Asian	519	272	383	86	49
Pacific Islander	3	0	7	0	0
Native American	16	8	17	4	0
NA	834	2664	745	141	137
Ethnicity					
Hispanic	269	294	479	189	73
Non-Hispanic	6438	3583	4730	1623	1642
NA	1096	2781	1239	169	172

*NSCLC= non-small cell lung cancer

Supplemental Table 8 (ST8). Demographic Characteristics of Select Metastatic Cancers in AACR GENIE: All Sites

Characteristic	*NSCLC	Breast	Colorectal	Pancreatic	Prostate
Total Number	4956	4215	3289	1399	1272
Race					
White	3622	3157	2389	1141	969
Black	367	322	258	65	99
Asian	351	213	144	65	40
Pacific Islander	4	2	0	0	1
Native American	7	3	5	3	0
NA	605	518	493	125	163
Ethnicity					
Hispanic	168	274	229	121	44
Non-Hispanic	4022	3341	2271	1116	1060
NA	766	600	789	162	168

*NSCLC= non-small cell lung cancer
